# Rapid detection of tetracycline resistance in bovine *Pasteurella multocida* isolates by MALDI Biotyper antibiotic susceptibility test rapid assay (MBT-ASTRA)

**DOI:** 10.1038/s41598-018-31562-8

**Published:** 2018-09-11

**Authors:** Laura Van Driessche, Jade Bokma, Linde Gille, Pieter-Jan Ceyssens, Katrin Sparbier, Freddy Haesebrouck, Piet Deprez, Filip Boyen, Bart Pardon

**Affiliations:** 1 Department of Large Animal Internal Medicine, Faculty of Veterinary Medicine, Ghent University, Salisburylaan 133, 9820 Merelbeke, Belgium; 2 National Reference Center for Tuberculosis and Mycobacteria, Scientific Institute of Public Health (WIV-ISP), Juliette Wytsmanstraat 14, 1050 Elsene, Belgium; 3 Bruker Daltonik GmbH, Fahrenheitstr. 4, 28359 Bremen, Germany; 4 Department of Pathology, Bacteriology and Avian Diseases, Faculty of Veterinary Medicine, Ghent University, Salisburylaan 133, 9820 Merelbeke, Belgium

## Abstract

*Pasteurella multocida* is notorious for its role as an opportunistic pathogen in infectious bronchopneumonia, the economically most important disease facing cattle industry and leading indication for antimicrobial therapy. To rationalize antimicrobial use, avoiding imprudent use of highly and critically important antimicrobials for human medicine, availability of a rapid antimicrobial susceptibility test is crucial. The objective of the present study was to design a MALDI Biotyper antibiotic susceptibility test rapid assay (MBT-ASTRA) procedure for tetracycline resistance detection in *P*. *multocida*. This procedure was validated on 100 clinical isolates with MIC-gradient strip test, and a comparison with disk diffusion was made. Sensitivity and specificity of the MBT-ASTRA procedure were 95.7% (95% confidence interval (CI) = 89.8–101.5) and 100% (95% CI = 100–100), respectively, classifying 98% of the isolates correctly after only three hours of incubation. Sensitivity and specificity of disk diffusion were 93.5% (95% CI = 86.3–100.6) and 96.3% (95% CI = 91.3–101.3) respectively, classifying 95% of the isolates correctly. In conclusion, this MBT-ASTRA procedure has all the potential to fulfil the need for a rapid and highly accurate tetracycline susceptibility testing in *P*. *multocida* to rationalize antimicrobial use in outbreaks of bronchopneumonia in cattle or other clinical presentations across species.

## Introduction

*Pasteurella multocida*, a Gram-negative coccobacillus, causes many important diseases in a wide range of hosts^1^. In different ruminant species and pigs it is one of the most frequently isolated pathogens in infectious bronchopneumonia^2,3^, a disease which is the leading cause of morbidity and antimicrobial use in these species^4^. In more tropical regions of the world, especially Africa and Asia, *P*. *multocida* can also cause highly fatal hemorrhagic septicemia in cattle and buffaloes^5^. In other animal species, this bacterium has been associated with presentations like sepsis^6^, but also otitis^7^ and peritonitis^8^. In humans, *P*. *multocida* can cause several life-threatening conditions, like sepsis^9^ and endocarditis^10^.

Today, to control bronchopneumonia mass medication is still one of the preferred therapies in different food-producing animal species^4,11,12^. Given the issue of high level antimicrobial resistance in these industries and the one health initiatives founded to combat this^13–15^, preventative antimicrobial use is no longer considered appropriate and antimicrobial treatment should be targeted^16^. In several European countries like Belgium, Germany or the Netherlands, guidelines suggest and legislation requires sampling and susceptibility testing of animal pathogens before specific antimicrobial agents can be used^17–23^. In addition, formularies have been initiated in several countries to guide veterinarians to select appropriate antimicrobials mainly based on their clinical efficacy and importance for human medicine^24,25^. Especially in more intensive cattle rearing systems which rely on mass medication, antimicrobial multiresistance in *P*. *multocida* is common, hampering empiric antimicrobial therapy^12,26–28^. Multiresistant isolates, that compromise 12 antimicrobial resistance genes, are threatening the future of these production systems^29^.

To date, mainly disk diffusion susceptibility tests are used in veterinary practice to guide antimicrobial therapy. These take a minimum of 2 days after sampling before results are available, but in most cases, due to different practical reasons, a waiting period of 3–4 days is average. In most outbreaks of infectious bronchopneumonia postponing an appropriate therapy, until susceptibility testing results are available, is not acceptable for economic and animal welfare reasons. On the other hand initiation of an inappropriate antibiotic treatment will increase antimicrobial resistance selection pressure^30^. Disk diffusion susceptibility tests have shown a sensitivity of only 85.7% for the detection of tetracycline resistance in *P*. *multocida* compared to the agar dilution technique as the gold standard in the past^31^, which might result to therapeutic failure in practice. The agar dilution technique is however not commonly used in routine veterinary diagnostics, being a more laborious and expensive technique compared with disk diffusion. Although the antimicrobial gradient strip method is not regarded as the gold standard like the broth dilution technique currently, results achieved by both tests have a good correlation^32–34^. Other advantages of the antimicrobial gradient strip test are the ease of use in combination with reliability and interpretability, resulting in a simple, near gold standard test. Since previous studies regarding new diagnostic techniques use this method for diagnostic accuracy, this was also performed as reference test in the current study.

Guidance of antimicrobial therapy in food animals is urgently needed, especially given the dense populations and potentially high disease incidences in the current production systems. Latter results in exposure of many animals, their bacteria and environment to antimicrobial selection pressure. To support veterinarians in their decision making process for antimicrobial therapy, not only highly accurate, but especially rapid diagnostic procedures are crucial. This to avoid unnecessary production loss, animal suffering or antimicrobial selection pressure.

The MALDI Biotyper antibiotic susceptibility test rapid assay (MBT-ASTRA) method, a MALDI-TOF MS-based approach for susceptibility testing, characterized by a semi-quantitative measurement of bacterial proteins, has shown promising results in obtaining rapid antimicrobial susceptibility results for human pathogens^35^. For some fast growing bacteria involved in sepsis in humans only 1–4 hours are needed for susceptibility testing^35–38^. Also for slow growers like mycobacteria this method seems viable^39^. Furthermore, this technique shows a high sensitivity and specificity compared to minimum inhibitory concentration (MIC)-gradient strip test^35–38^. The potential of MBT-ASTRA for veterinary indications, especially those associated with mass medication, has not been explored. Furthermore applying this method on fastidious growers like *P*. *multocida* or any other *Pasteurellaceae* has not yet been described.

Therefore, the primary objective of the present study was to design and validate an MBT-ASTRA procedure for tetracycline resistance detection in *P*. *multocida* from bovine origin. The secondary objective was to determine diagnostic accuracy of this MBT-ASTRA procedure and classic disk diffusion compared to MIC-gradient strip testing.

## Results

### Determination of standard testing conditions for MBT-ASTRA

**Figure 1:**
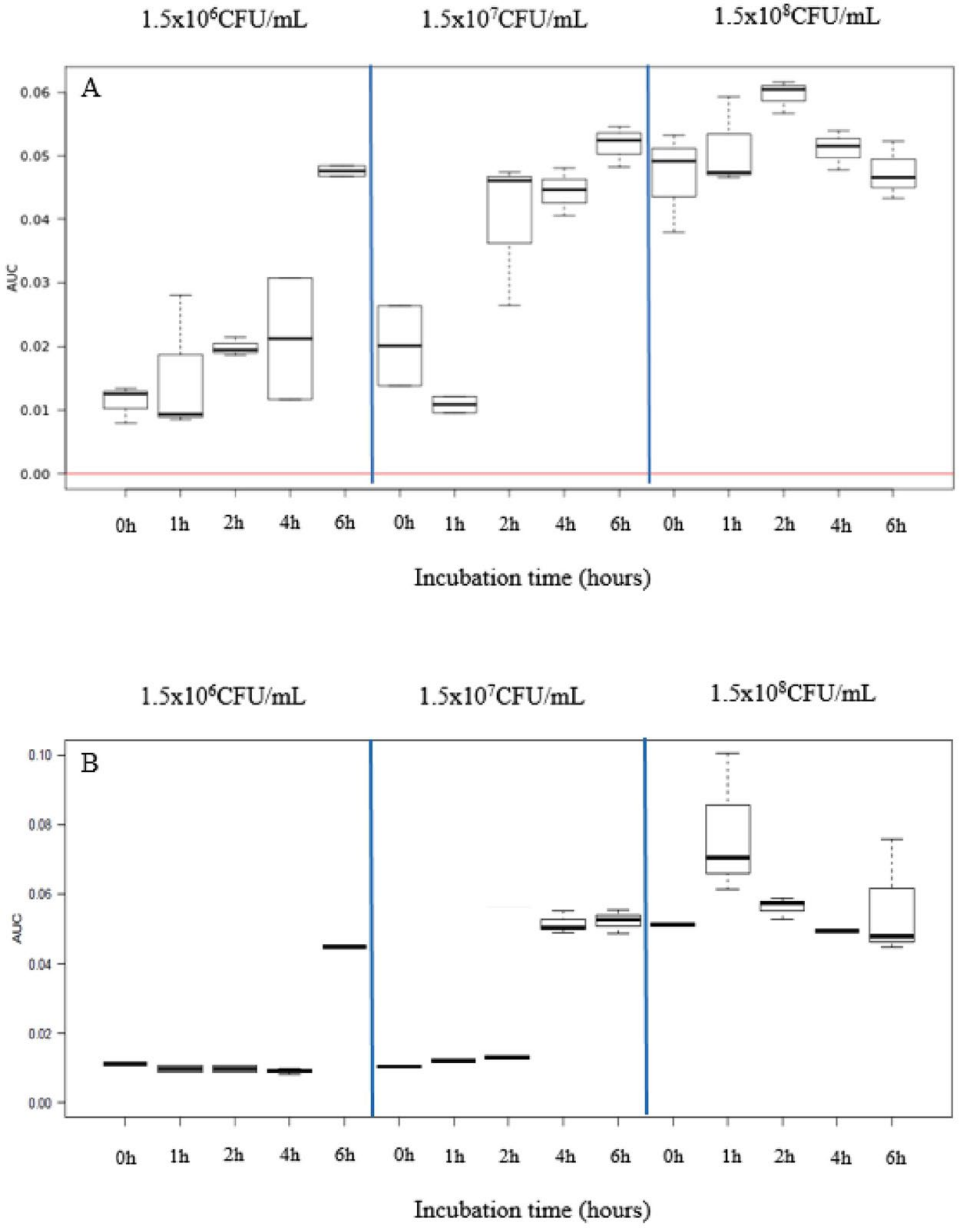
Comparison of CAMHB (**A**) and BHIB (**B**) medium and different *P*. *multocida* starting concentrations to optimize bacterial growth to allow identification in an MBT-ASTRA procedure. Presented spectra are representative for the repetitions made using a single *P*. *multocida* isolate (P114, MIC: 0.19 µg/mL).

To determine the optimal growth medium and starting concentration of the bacterial suspension, one susceptible isolate (P114: MIC: 0.19 µg/mL) was incubated in two different media using three different starting concentrations for incubation times varying between 0 and 6 hours (Fig. 1). This experiment was repeated two times. No difference in growth, expressed by the area under the curve (AUC), was seen between Cation-adjusted Mueller Hinton broth (CAMHB) and Brain Heart Infusion broth (BHIB) (Fig. 1). Starting concentration clearly affected growth, reaching sufficient bacterial growth for MALDI-TOF identification after 6, 2–4 and 0 h with 1.5 × 10^6^ CFU/mL, 1.5 × 10^7^ CFU/mL and 1.5 × 10^8^ CFU/mL, respectively (Fig. 1).

**Figure 2:**
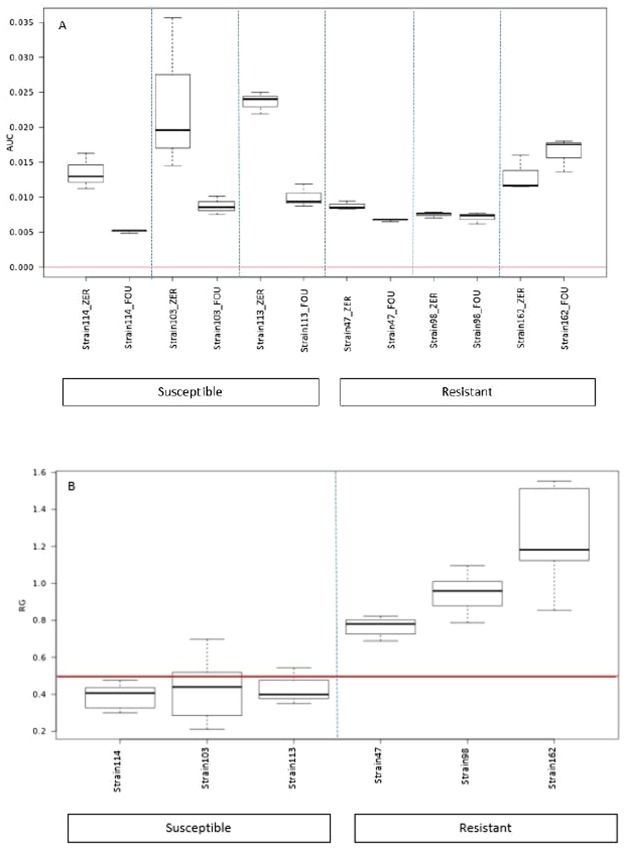
Area under the curve (AUC) (**A**) and Relative Growth (RG) (**B**) box plots of 3 susceptible and 3 resistant *P*. *multocida* isolates after 3 hours of incubation without antibiotic (=ZER) or with 4 µg/mL of tetracycline (=FOU). For resistant strains, no clear difference between both AUCs is noticed and RGs are high, whereas susceptible strains obtain a lower RG. The horizontal red line represents a RG cut-off value of 0.5.

Second, the antibiotic concentration allowing a clear separation between resistant and susceptible isolates was determined. To obtain this value, three susceptible (P114; MIC 0.19 µg/mL, P103; MIC 0.25 µg/mL and P113; MIC 0.5 µg/mL) and three resistant (P47; MIC 16 µg/mL, P162; MIC 24 µg/mL and P98; MIC 48 µg/mL) isolates of *P*. *multocida* were tested with tetracycline concentrations of 0, 2, 4 and 8 µg/mL and were incubated for 0, 3, 4, 5 and 6 hours. This experiment was performed 3 times independently, each using 1 resistant and 1 susceptible strain. After incubation, the growth derived from an increased protein signal of these isolates was calculated by measuring the AUC of the obtained spectrum. In the susceptible isolates, the AUC of the sample with antibiotic should be significantly reduced in comparison with the sample without antibiotic. For resistant isolates, no significant difference in AUC should be noticed. The relative growth (RG) ratio is the ratio of the AUC derived from spectra with and without antibiotic and is used to distinguish susceptible from resistant isolates according to an empirical cut-off value. A clear visual difference between susceptible and resistant isolates was achieved at a tetracycline concentration of 4 µg/mL and a RG cut-off value of 0.5 (Fig. 2). In this preliminary test, the three resistant isolates showed a RG > 0.5, while the susceptibile isolates showed a RG < 0.5. A correct classification of all strains was already possible after 3 hours of incubation with 4 µg/mL tetracycline (Fig. 2).

### Determination of the diagnostic accuracy of the MBT-ASTRA procedure and comparison with the disk diffusion method

#### MIC distribution of the study population

**Figure 3:**
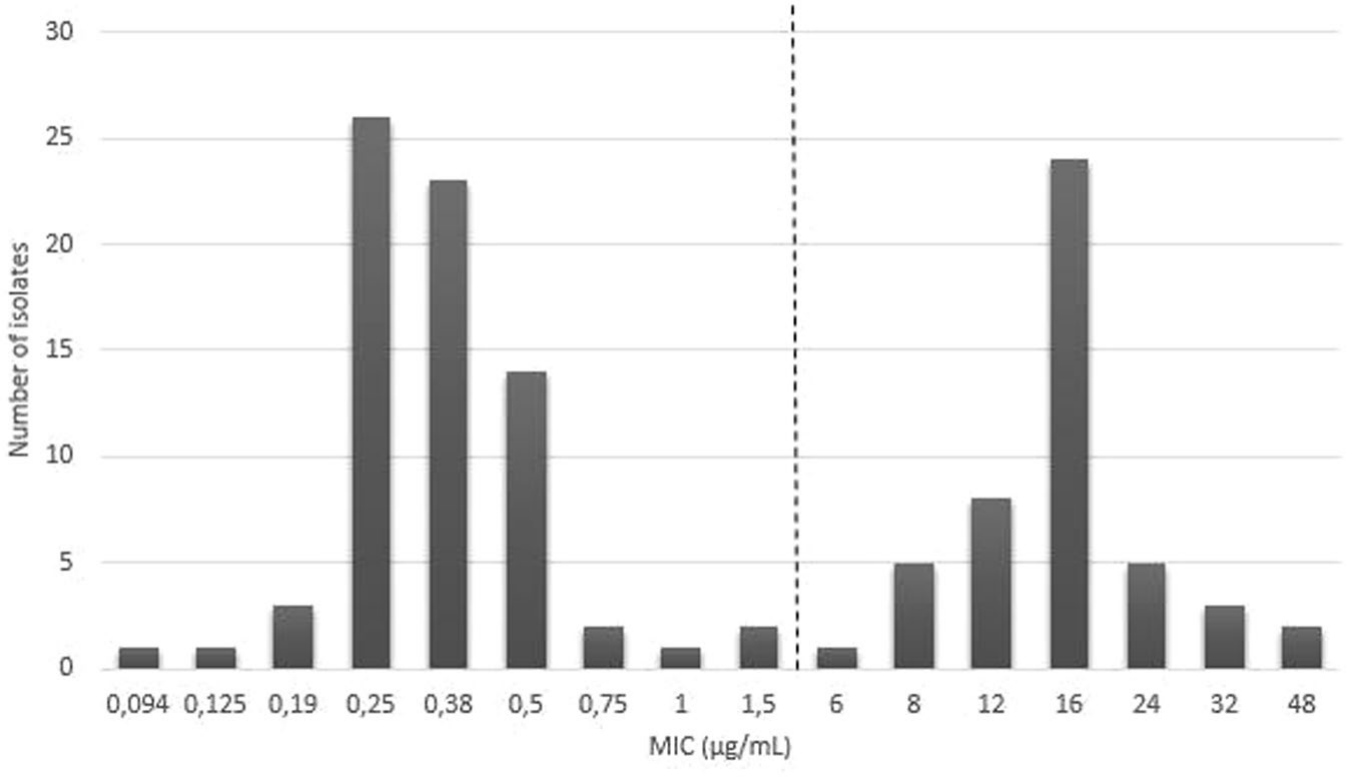
MIC values for tetracycline of 100 recent clinical isolates of *P*. *multocida* used to determine diagnostic accuracy of an MBT-ASTRA procedure for this antimicrobial-bacterium combination. MIC values were determined by the MIC-gradient strip test. The vertical line represents the CLSI clinical breakpoint for susceptibility (≤2 µg/mL)^40^.

A total of 100 clinical *P*. *multocida* isolates were used to validate the MBT-ASTRA with the MIC-gradient strip test as reference test used in the current study. Of the isolates tested, 54 and 46 were classified as susceptible or resistant by MIC-gradient strip test, respectively. This experiment was conducted once. Tetracycline MIC-values for the quality control reference strains were tested twice and were in the acceptable range according to CLSI standards^40^, i.e. *Staphylococcus aureus* ATCC 29213 MIC-value 0.38 µg/mL and 0.5 µg/mL (range 0.12–1 µg/mL), *Escherichia coli* ATCC 25922 MIC-value 0.5 µg/mL and 1.5 µg/mL (range 0.5–2 µg/mL) and *Enterococcus faecalis* ATCC 29212 MIC-value 16 µg/mL (range 8–32 µg/mL). The tested isolates showed a bimodal MIC distribution, ranging between 0.094 and 48 µg/mL (Fig. 3). According to veterinary CLSI standards^40^, *P*. *multocida* isolates are considered responsive to treatment (=susceptible) with tetracycline in cattle when they show an MIC value ≤ 2 µg/mL.

#### Diagnostic accuracy of MBT-ASTRA and disk diffusion

**Figure 4:**
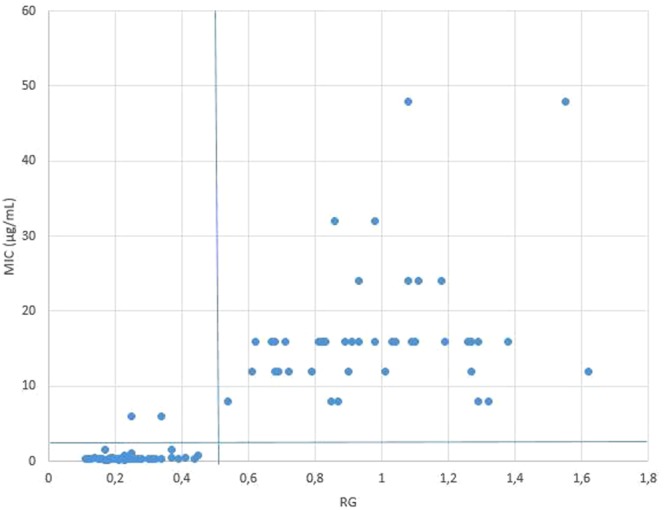
Scatter plot of minimum inhibitory concentrations (MIC) obtained with the MIC-gradient strip test with MBT-ASTRA relative growth (RG) values of 100 recent bovine clinical strains of *P*. *multocida*. MBT-ASTRA testing conditions were 3 hours of incubation with a concentration of tetracycline of 4 µg/mL. The horizontal and vertical line represent the clinical breakpoint of ≤2 µg/mL and the RG cut-off value of 0.5, respectively. Two isolates with an MIC value of 6 µg/mL are considered susceptible with MBT-ASTRA, causing 2 false susceptible results.

The MBT-ASTRA procedure was conducted for 100 clinical isolates with an incubation time of 3 hours, with and without a tetracycline concentration of 4 µg/mL in CAMHB. This test was performed once. Relative growth values were determined for all tested isolates. Receiver operating characteristics (ROC) curve analysis showed a relative growth value of 0.5 to be the optimal cut-off to differentiate resistant from susceptible isolates for this MBT-ASTRA method (Fig. 4).

For the disk diffusion tests, quality control reference strains were included and inhibition zones (in mm) were in the acceptable range according to CLSI standards^40^, i.e. *Staphylococcus aureus* ATCC 25923 inhibition zone 24 mm (range 24–30 mm) and *Escherichia coli* ATCC 25922 inhibition zone 22 mm (range 18–25 mm). The disk diffusion method with quality control reference strains included was performed once.

Table 1 shows classification of the test results of both MBT-ASTRA and disk diffusion compared to the MIC-gradient strip test. At a RG cut-off value of 0.5, a correct classification of all 54 susceptible strains and 95.7% (44/46) of resistant strains was achieved. All susceptible isolates showed RG values smaller than 0.5. All resistant isolates had RG values above 0.5, except two isolates (P33, P106) with an MIC value of 6 µg/mL that showed a RG value of 0.34 and 0.25 respectively, causing 2 false susceptible results (Fig. 4).

**Table 1:** 2 × 2 Contingency table showing tetracycline resistance testing results of MBT-ASTRA and disk diffusion compared to MIC-gradient strip test for 100 recent clinical *P. multocida* isolates derived from cattle.

Test		Reference test (MIC-gradient strip test)	
		*Resistant*	*Susceptible*
MBT-ASTRA	*Resistant*	95.7% (44/46)	0% (0/54)
	*Susceptible*	4.3% (2/46)	100% (54/54)
	*Total*	100% (46/46)	100% (54/54)
Disk diffusion	*Resistant*	93.5% (43/46)	3.7% (2/54)
	*Susceptible*	6.5% (3/46)	96.2% (52/54)
	*Total*	100% (46/46)	100% (54/54)

Diagnostic accuracy of the disk diffusion and MBT-ASTRA method was calculated using the MIC-gradient strip test as reference (Table 2). Compared to MIC-gradient strip testing, the MBT-ASTRA method achieved a sensitivity and specificity of 95.7% and 100%, respectively. Sensitivity and specificity of disk diffusion were 93.5% and 96.3%, respectively. The essential agreement of MBT-ASTRA with the MIC-gradient strip test is 98%, due to 2% very major errors (Table 2). In contrast, the disk diffusion method obtained an essential agreement of 95%, including 3% very major errors and 2% major errors (Table 2).

**Table 2:** Diagnostic accuracy of disk diffusion and MBT-ASTRA for tetracycline susceptibility testing in 100 clinical *P*. *multocida* isolates from cattle compared to the MIC-gradient strip test.

	Disk diffusion	MBT-ASTRA
*Essential agreement*	95%	98%
*Very major error*	3%	2%
*Major error*	2%	0%
*Minor error*	0%	0%
*Sensitivity*	93.5%	95.7%
	(86.3%, 100.6%)	(89.8%, 101.5%)
*Specificity*	96.3%	100%
	(91.3%, 101.3%)	(100.0%, 100.0%)
*RPV*	95.6%	100%
	(89.5%, 101.6%)	(100.0%, 100.0%)
*SPV*	94.5%	96.4%
	(88.5%, 100.5%)	(91.6%, 101.3%)

Values between brackets represent the 95% confidence interval of the estimate. Definitions describing the diagnostic accuracy^31,47,48^.

Essential agreement: results of both techniques identical; very major: resistant strain by the MIC-gradient strip test method misinterpreted as susceptible by disk diffusion/MBT-ASTRA; major: susceptible strain by the MIC-gradient strip test method misinterpreted as resistant by disk diffusion/MBT-ASTRA; minor error: intermediate result was obtained by only one method; sensitivity is number of resistant strains by disk diffusion or MBT-ASTRA/number of resistant strains by MIC-gradient strip test; specificity is number of susceptible strains by disk diffusion or MBT-ASTRA/number of susceptible strains by MIC-gradient strip test. RPV, resistant (positive) predictive value; the probability that a strain is truly resistant if the disk diffusion method or MBT-ASTRA categorizes a strain as resistant; SPV, susceptible (negative) predictive value is defined as the probability that a strain is truly susceptible if the disk diffusion method or MBT-ASTRA categorizes a strain as susceptible.

## Discussion

This study aimed at developing a MALDI-TOF MS-based approach for rapid tetracycline susceptibility testing in *P*. *multocida* and to compare this technique and the current standard test in practice (disk diffusion) with the MIC-gradient strip test. The most widely accepted gold standard for susceptibility testing in bacteria is the broth dilution technique^41,42^. In order to compare the obtained data with previous studies, which commonly used the MIC-gradient strip test, the authors also opted for MIC-gradient strip testing^35–38^. MIC-gradient strip test results have a good correlation with MIC values achieved by broth dilution technique, and is often considered as a near gold standard test^32–34^. Therefore, the authors believe that current results based on the MIC-gradient strip test will hardly differ from broth dilution results. Further research can include latter technique as gold standard for determining the accuracy of the MBT-ASTRA method.

Another limitation was that no intermediate results were obtained from neither techniques. In practice, to avoid therapeutic failure, intermediate results are often regarded as resistant, and another antimicrobial is selected. As the MBT-ASTRA method is currently unable to correctly classify intermediate susceptibility isolates for a given antibiotic, including isolates with intermediate results on the reference test would have been an added value. Unfortunately, no isolates classified as intermediate by MIC-gradient strip test were obtained in this study. Also in previous work, this has not been accounted for^36,37^. Modifications to this method that would allow classification of intermediate include correlations between the relative growth and MIC value and by using a serial dilution of the given antibiotic to define an MBT-ASTRA minimal breakpoint^37^. However, classification as intermediate is contra productive for clinical decision making and may lead to more inappropriate treatments.

The main conclusion of the present study is that the MBT-ASTRA method allows for a tetracycline susceptibility testing result for *P*. *multocida* in only three hours of incubation with high accuracy. This incubation time is in the same range compared to the MBT-ASTRA method for fast growing bacteria involved in sepsis in humans^35–38^. Although *P*. *multocida* is considered a fastidiously growing microorganism by CLSI^40^ and tetracycline is considered bacteriostatic, which might cause a longer incubation time to obtain a clear difference in AUC with or without antibiotic. Due to this short incubation period, the MBT-ASTRA method makes it possible to provide the results of the susceptibility test on the same day of identification, avoiding unnecessary production loss or animal suffering. Sensitivity (95.7%) and specificity (100%) of the presented MBT-ASTRA procedure were excessive, in the range of and even higher than compared to previous findings applying MBT-ASTRA for other species under different conditions^35–38^. In contrast, the disk diffusion method evaluated in this study had lower sensitivity (93.5%) and specificity (96.3%). A previous study on *P*. *multocida* isolates showed lower sensitivity (85.7%) and higher specificity (99.1%) of disk diffusion for tetracycline resistance detection^31^. A possible explanation for this difference could be that at that time disks with higher tetracycline contents (80 µg) were used and clinical breakpoints differed with those obtained in the current study. Classification of the 100 isolates with disk diffusion was performed using the interpretative criteria of the manufacturer’s guidelines. Currently, no *P*. *multocida*-specific clinical breakpoints for tetracycline in cattle are available for disk diffusion, while they are described for MIC testing^40^. Zone diameters (mm) were measured and analysed. A clear difference between susceptible (zone diameter ranging from 24–32 mm) and resistant (zone diameter ranging from 8–10 mm) isolates was noticed. Due to these considerable differences in zone diameter, modifying the clinical breakpoints would have little to no effect on the results.

Compared to the disk diffusion method as used under the current circumstances, MBT-ASTRA shows less discrepancies to the MIC-gradient strip test method. However, in 2% of the isolates tested a very major error was present, potentially resulting in treatment of the animal with tetracycline when the isolate is resistant. Both of these isolates had an MIC value of 6 µg/mL. According to CLSI standards, an MIC value of 4 µg/mL is classified as intermediate and an MIC value ≥ 8 µg/mL is classified as resistant. Considering these clinical breakpoints, both strains with an MIC value of 6 µg/mL can be classified as (borderline) resistant, resulting in 2% very major errors and a sensitivity of 95.7% with the MBT-ASTRA method. However, the clinical outcome of treatment with tetracycline of *P*. *multocida* isolates with an MIC value of 6 µg/mL might be difficult to distinctly predict.

Given its rapid results and high diagnostic accuracy, this MBT-ASTRA technique has high potential to be used in the field as a decision aid to direct antimicrobial use in food animals. In order to fulfil this potential, the possibilities of obtaining a full susceptibility test (as many antimicrobials tested at once as in disk diffusion) need to be explored, and the practical issue of availability of a MALDI-TOF MS in the regional laboratories needs to be overcome. However, in recent years the use of MALDI-TOF MS has been advanced to a standard method in clinical microbiology laboratories, due to its rapid and low-cost characteristics^43^. Considering the simple setup and short incubation time, the MBT-ASTRA method implements an early intervention of adequate antibiotic therapy, resulting in a cost-and –labour efficient tool like the disk diffusion method. Whether the current MBT-ASTRA settings are also applicable to other *Pasteurellaceae* of clinical importance in veterinary medicine, like *Mannheimia haemolytica* or *Histophilus somni*, needs to be determined.

In conclusion, the MBT-ASTRA method developed in the present study, has all the potential to fulfil the need for a rapid and highly accurate tetracycline susceptibility testing in *P*. *multocida*. This is essential to rationalize antimicrobial use in outbreaks of bronchopneumonia in cattle or other clinical presentations across species.

## Methods

### Determination of the standard conditions for MBT-ASTRA

#### Selection of the optimal growth medium and starting concentration of P. multocida

BHIB (Difco, BD Diagnostic Systems, Sparks, Md.) and CAMHB (Difco, BD Diagnostic Systems, Sparks, Md.) were used as growth media in this study. One susceptible strain of *P*. *multocida* (P114, MIC 0.19 µg/mL) was inoculated in 10 mL of BHIB or 10 mL of CAMHB at different starting concentrations. Starting concentrations tested in both media were 1.5 × 10^6^ CFU/mL, 1.5 × 10^7^ CFU/mL and 1.5 × 10^8^ CFU/mL. No antibiotics were added to the medium. All tubes were placed in a shaking incubator for 0, 1, 2, 3, 4 and 6 hours at a temperature of 37 °C and an atmosphere enriched with 5% CO_2_. After each incubation period, 1 mL samples of each tube were transferred to Eppendorf tubes.

#### Selection of the optimal tetracycline concentration and incubation time

To determine the optimal concentration of tetracycline and the shortest incubation time necessary to differentiate between susceptible and resistant isolates, three susceptible isolates (P114; MIC 0.19 µg/mL, P103; MIC 0.25 µg/mL, P113; MIC 0.5 µg/mL) and three resistant isolates (P47; MIC 16 µg/mL, P162; MIC 24 µg/mL and P98; MIC 48 µg/mL) were used. Tetracycline concentrations of 0, 2, 4 and 8 µg/mL were tested (Sigma-Aldrich, Germany) at incubation periods of 0–3–4–5 and 6 hours.

#### MBT-ASTRA sample preparation

Eppendorf tubes, containing 1 mL samples grown for the indicated incubation time, were centrifuged at 21130 × g for 5 minutes at room temperature. After centrifugation, the supernatant was carefully aspirated and 700 µL of 70% ethanol in high performance liquid chromatography (HPLC) graded water was added to the cell pellet and vortexed. A second centrifugation and aspiration of the supernatant was performed as mentioned above. After air drying for a minimum of 10 minutes, the cell pellets were stored at −20 °C for up to a maximum of 3 days. After storing, 20 µL 70% formic acid (in HPLC graded water) was added to the cell pellet and mixed carefully. Samples were incubated for five minutes at room temperature. In a last step, 20 µL of acetonitrile was added and vortexed. Before adding acetonitrile to the cell pellet, an internal standard^35^ (Bruker Daltonik GmbH, Bremen, Germany, suspended in 25 µL HPLC water) was added to acetonitrile (ratio of 1/100, using 0.2 µL of internal standard in 20 µL of acetonitrile per sample) for spectra acquisition and facilitating the semi-quantitative analysis of the acquired spectra to quantify the difference in biomass corresponding to the growth between different setups. A third centrifugation step (21130 × g for 2 minutes) was performed to clarify the lysates.

#### MALDI-TOF MS analysis

One µL of the protein extraction was spotted in triplicate on the target plate (MSP 96 target polished steel BC). After air drying of the spots, 1 µl of matrix (10 mg/ml of α-cyano-4-hydroxy-cinnamic acid [α-HCCA] in 50% acetonitrile - 47.5% water - 2.5% trifluoroacetic acid; Bruker Daltonik GmbH, Bremen, Germany) was placed on each spot. External calibration was included in each measurement using a bacterial test standard (BTS, Bruker Daltonik GmbH, Bremen, Germany). Analysis was performed with an Autoflex III smartbeam MALDI-TOF MS instrument (Bruker Daltonik GmbH, Bremen, Germany), recording the mass range between 2.000–20.000 Da using standard settings. Automated data analysis was performed with the MBT-ASTRA software prototype written in the software package R^35^. A mass range of 2.500–13.500 was used for analysis. AUC and relative growth rate were calculated automatically for each setup. The duration of MALDI-TOF analysis is around 10–15 minutes for one sample. This duration is not cumulative when analysing multiple samples.

The obtained optimal test conditions were subsequently used during assay validation, analysing 100 recent clinical *P*. *multocida isolates* to determine the cut-off value of the relative growth ratio that allows a differentiation between susceptible and resistant isolates.

### Determination of diagnostic accuracy of the MBT-ASTRA procedure and comparison with the disk diffusion method

#### Bacterial isolates and cultivation

In this experiment, 100 isolates of *P*. *multocida* from calves with bronchopneumonia collected in Belgium between 2014 and 2017 were analysed. Sampling of these calves was performed using a deep nasopharyngeal swab or a broncho-alveolar lavage as previously described^44^. All methods were carried out in accordance with relevant guidelines and regulations. All experimental protocols were approved by the ethical committee of the Faculty of Veterinary Medicine, Ghent University (EC 2014/164, EC 2016/20). Identification of *P*. *multocida* isolates was first achieved by standard biochemical tests^45^ and subsequently confirmed with MALDI-TOF MS using the direct transfer protocol as previously described^46^. Bacteria were cultivated overnight at 37 °C and an atmosphere enriched with 5% CO_2_ on Columbia agar (Oxoïd, UK) supplemented with 5% sheep blood.

Three susceptibility testing methods were performed on all isolates, namely MIC-gradient strip test, which is comparable to the standard dilution test^32–34^, the MBT-ASTRA procedure as described above and disk diffusion as the current standard test in practice.

#### MIC-gradient strip test

The MIC values of tetracycline were determined using MIC-gradient strips. Briefly, MIC test strips (Liofilchem, Italy) were placed on Mueller Hinton agar plates enriched with 5% defibrinated sheep blood (BD Diagnostics Systems), shortly after the plates had been uniformly inoculated via polyester swabs with 0.5 McFarland suspensions of the pure isolates. Incubation was performed for 18–24 hours under aerobic conditions at 35 °C. *Staphylococcus aureus* ATCC 29213 and *Escherichia coli* ATCC 25922 were used as quality control reference strains. MIC values were determined according to the manufacturer’s instructions. Determination of susceptibility was performed according to the current CLSI standards for bovine *P*. *multocida* (i.e. susceptible ≤2 µg/mL, intermediate 4 µg/mL, resistant ≥8 µg/mL)^40^. Used media, incubation conditions, quality control strains and used interpretive criteria were applied according to CLSI standards^40^. Inoculation method was performed according to manufacturer’s guidelines.

#### MBT-ASTRA procedure

All isolates were incubated for 3 hours with and without a tetracycline concentration of 4 µg/mL in CAMHB with a starting concentration of 1.5 × 10^7^CFU/mL. MBT-ASTRA sample preparation and MALDI-TOF MS analysis were performed immediately after incubation as mentioned above.

#### Disk diffusion test

Susceptibility testing of *P*. *multocida* and tetracycline was performed in accordance with CLSI standards^40^. Briefly, Mueller Hinton agar (Oxoïd, UK) plates, supplemented with 5% sheep blood, were uniformly inoculated via polyester swabs with 0.5 McFarland suspensions of the pure isolates. Then, tetracycline disks (30 µg/mL, Rosco, Neosensitabs, Taarstrup, Denmark) were placed on Mueller Hinton agar (Oxoïd, UK) supplemented with 5% sheep blood, shortly after the plates had been uniformly inoculated via polyester swabs with 0.5 McFarland suspensions of the pure isolates. Incubation was performed for 18–24 hours at 35 °C in ambient air. Analysis of inhibition zones (in mm) was performed and interpretative criteria were according to manufacturer’s guidelines. *Staphylococcus aureus* ATCC 25923 and *Escherichia coli* ATCC 25922 were used as quality control reference strains.

#### Statistical analysis

Receiver operating characteristics (ROC) curve analysis was used to determine the optimal cut-off of the relative growth ratio to distinguish resistant from susceptible isolates in the MBT-ASTRA procedure (SPSS statistics vs. 24 (IBM, New York, United States)). Performance of the MBT-ASTRA and disk diffusion method compared to the MIC-gradient strip test as the gold standard was determined. Results were presented by distinguishing very major (resistant strain by the MIC-gradient strip test method misinterpreted as susceptible by the disk diffusion/MBT-ASTRA method), major (susceptible strain by the MIC-gradient strip test method misinterpreted as resistant by the disk diffusion/MBT-ASTRA method) and minor errors (intermediate result was obtained by only one method)^31,47,48^. Diagnostic accuracy, sensitivity, specificity, RPV and SPV were derived from 2 × 2 contingency tables using Winepiscope 2.0^49^.

## Data Availability

The authors declare that all information obtained from this study is presented in this paper.
